# The Mitochondrial HSP90 Paralog TRAP1: Structural Dynamics, Interactome, Role in Metabolic Regulation, and Inhibitors

**DOI:** 10.3390/biom12070880

**Published:** 2022-06-24

**Authors:** Abhinav Joshi, Takeshi Ito, Didier Picard, Len Neckers

**Affiliations:** 1 Urologic Oncology Branch, Center for Cancer Research, National Cancer Institute (NCI), Bethesda, MD 20892, USA; aj.joshi@nih.gov (A.J.); takeshi.ito@nih.gov (T.I.); 2 Department of Molecular and Cellular Biology, Université de Genève, Sciences III, 30 Quai Ernest-Ansermet, CH-1211 Geneva, Switzerland; didier.picard@unige.ch

**Keywords:** HSP90, TRAP1, molecular chaperone, mitochondria, metabolism, OxPhos, tetramers

## Abstract

The HSP90 paralog TRAP1 was discovered more than 20 years ago; yet, a detailed understanding of the function of this mitochondrial molecular chaperone remains elusive. The dispensable nature of TRAP1 in vitro and in vivo further complicates an understanding of its role in mitochondrial biology. TRAP1 is more homologous to the bacterial HSP90, HtpG, than to eukaryotic HSP90. Lacking co-chaperones, the unique structural features of TRAP1 likely regulate its temperature-sensitive ATPase activity and shed light on the alternative mechanisms driving the chaperone’s nucleotide-dependent cycle in a defined environment whose physiological temperature approaches 50 °C. TRAP1 appears to be an important bioregulator of mitochondrial respiration, mediating the balance between oxidative phosphorylation and glycolysis, while at the same time promoting mitochondrial homeostasis and displaying cytoprotective activity. Inactivation/loss of TRAP1 has been observed in several neurodegenerative diseases while TRAP1 expression is reported to be elevated in multiple cancers and, as with HSP90, evidence of addiction to TRAP1 has been observed. In this review, we summarize what is currently known about this unique HSP90 paralog and why a better understanding of TRAP1 structure, function, and regulation is likely to enhance our understanding of the mechanistic basis of mitochondrial homeostasis.

## 1. Introduction

Molecular chaperones form one of the central pillars of the cellular proteostasis network [1,2]. Depending upon their function, these molecules fall into three fundamental classes: foldases, holdases, and disaggregases [1,2,3,4,5,6,7]. Under certain circumstances, some molecular chaperones also deliver damaged and impossible to fold client proteins for degradation by proteosomes or autophagy [8,9,10]. Foldases are ATP-dependent chaperones that actively fold nascent proteins into their native functional conformations and refold unfolded proteins under cellular stress. Heat shock protein 90 (HSP90) is an ATP-dependent foldase that is remarkably conserved from bacteria to humans [11]. It regulates folding, maturation, and stability of proteins (in HSP90’s case, termed “clients”) that are involved in cell growth, survival, apoptosis, and adaptation to stress [12,13,14].

In mammalian cells there are four different HSP90 paralogs: HSP90α, HSP90β, GRP94, and TRAP1. HSP90α and HSP90β are primarily cytosolic with a small component in the nucleus. HSP90α is stress induced while HSP90β is constitutively expressed [15]. GRP94 is localized in the endoplasmic reticulum [16] and TRAP1 (or HSP75), the paralog on which we focused in this review, is primarily localized in mitochondria [17,18]. TRAP1 was initially identified in 2000 [17] and was widely presumed to facilitate late-stage folding of clients in the mitochondrial matrix. However, it increasingly became clear that this may not be the case. TRAP1 has since been implicated in metabolic regulation [19,20,21,22,23,24,25], mitochondrial dynamics [26], mitophagy [27,28], protection from oxidative stress [23,29,30,31,32], and protection from cell death [33].

## 2. TRAP1: Cytoprotective or Pro-Neoplastic?

Although TRAP1 may have regulatory roles in organellar processes, whether it is ultimately cytoprotective in the context of neurodegenerative diseases or pro-neoplastic in the context of many cancers may reflect two sides of the same coin. This molecule has been reported to play a crucial role in inhibiting oxidative stress-induced tissue damage in the ischemic brain [34], hypoxia-induced injury in cardiomyocytes [35], myocardial ischemia/reperfusion injury [36], motor neuron degeneration in oxidative stress-induced amyotrophic lateral sclerosis (ALS) [37], and acidosis-induced injury in cardiomyocytes [38]. Likewise, TRAP1 appears to be protective in genetic models of neurodegeneration such as Parkinson’s disease [27,28,39] where protein quality control in mitochondria plays a critical role [40]. TRAP1 was also shown to be mitoprotective in models of kidney fibrosis and renal cell carcinoma [41,42]. Finally, loss-of-function TRAP1 mutations have been identified in the brain of a patient with Parkinson’s disease [43], Leigh syndrome [44], and chronic functional symptomatology including pain, fatigue, and gastrointestinal dysmotility [45], and in congenital abnormalities associated with the kidney (CAKUT) [46].

While these studies identify TRAP1 as cytoprotective in mitochondrial-associated neuropathologies, other studies have highlighted a potential pro-neoplastic role of TRAP1 in cancer, where it can also display cytoprotective and other pro-tumorigenic activities. Thus, TRAP1 expression was found to be increased in hepatocellular carcinoma [47], breast cancer [48], glioma [49], small cell lung cancer [50], and kidney, prostrate, ovarian, colorectal, and esophageal cancer, and it is correlated with advanced-stage metastatic tumors with poor prognosis [51,52,53,54,55,56,57]. In colorectal cancer and its animal models, increased TRAP1 expression was found to be localized to pro-neoplastic lesions in the tumor [58,59]. While data supporting the importance of TRAP1 are numerous [24,47,48,49,50,51,52,53,54,55,56,57,59,60], these findings are challenged by other reports where TRAP1 expression inversely correlates with tumor stage [19] or is seemingly unimpactful in carcinogenesis models in TRAP1 knockout (KO) mice [61]. This has led to a general consensus that TRAP1’s role may be more context dependent.

Nevertheless, TRAP1 does appear to play a role in the metabolic adaptation that may sustain neoplastic growth in a nutrient- and oxygen-poor environment; this hypothesis has driven research to mechanistically elucidate a role played by TRAP1 that is common to various cancers. Thus, TRAP1 was reported to play a critical role in the metabolic switch from oxidative phosphorylation (OxPhos) to aerobic glycolysis [19]. This relationship of TRAP1 to metabolic plasticity sparked an interest in exploring the details of TRAP1 structure, interactome, mode of action, and inhibitors. The data that has emerged since has definitively highlighted TRAP1 as a major player in mitochondrial bioenergetics. In this review, we hoped to provide a foundation for understanding the importance of TRAP1 in modulating mitochondrial homeostasis and the balance between oxidative phosphorylation and glycolysis.

## 3. Structure, ATPase Cycle, Dimers, and Tetramers

The TRAP1 gene is evolutionarily conserved [62] and is found in both metazoans and protozoans but not in the budding yeast. Unlike HSP90, TRAP1 is not an essential protein, and TRAP1 KO mice or cells derived therefrom are viable [19,63]. Likewise, loss of TRAP1 function in a patient with Parkinson’s disease was unimpactful [43]. Similar to all members of the HSP90 family, TRAP1 has been primarily reported to form and function as a homodimer, with each protomer being comprised of an N-terminal ATPase domain (NTD), a middle domain (MD), and a C-terminal dimerization domain (CTD) [11,64,65]. The N-terminal domain contains a 59-amino acid mitochondrial-targeting sequence that is cleaved upon import [66]. Interestingly, TRAP1 more closely resembles bacterial HSP90 (HtpG) than human HSP90 [17,67]. As with HtpG, but unlike HSP90, TRAP1 lacks both a charged linker domain between the NTD and MD and a C-terminal EEVD motif that serves as a co-chaperone interaction domain in HSP90. TRAP1 also features an extended β-strand in the NTD, called “strap”, that facilitates a cross protomer interaction in *trans* in the closed state of TRAP1. Removal of the “strap” domain dramatically upregulates ATPase activity; this extension is considered to be involved in the thermoregulation of the TRAP1 ATPase and to be potentially inhibitory for TRAP1 function under low temperatures [68].

TRAP1 is a nucleotide-dependent and nucleotide-activated chaperone that exists as a coiled-coil dimer in an autoinhibited state in the absence of ATP [69]. The presence of ATP activates the TRAP1 homodimer, which cycles between an open “apo” state and a closed state involving a series of ATP-dependent steps that promote large conformational changes within the molecule [70]. Unlike the rest of the HSP90 family, TRAP1 has a unique ATP-bound catalytically active state that adopts a strained asymmetric conformation [71]. This unique asymmetry is most pronounced in the highly conserved client binding region and results from the buckling of one of the protomers onto the other [71]. Interestingly, ATP hydrolysis is sequential between the two protomers, with the dimer undergoing a “flip” in the asymmetry while still remaining in the closed state [71]. The first ATP hydrolysis step facilitates client folding while the second leads to client unloading and return to an apo state [68]. The Mg^2+^ ion is the primary choice of cofactor for the TRAP1 ATPase, but it can be replaced by Ca^2+^ [72]. Surprisingly, Ca^2+^-bound TRAP1 displays cooperative ATP hydrolysis and avoids asymmetric flipping of its protomers [72]. This may indicate that TRAP1 can function both as a foldase and a holdase, depending on its ionic environment.

**Figure 1:**
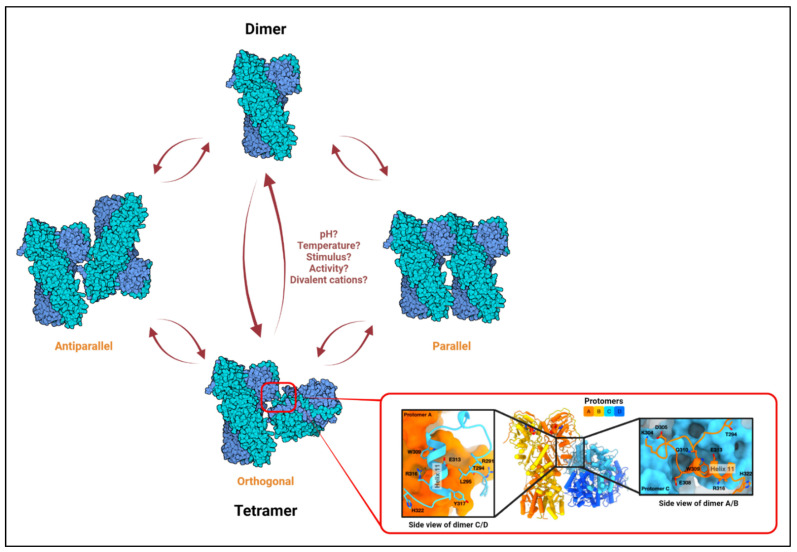
The TRAP1 tetramer. Based on in vitro studies on HSP90 oligomerization, rapid alterations in temperature, chaperone activity, or local concentration of divalent cations, which are common occurrences in the mitochondria, may influence dimer–tetramer transition. Three distinct conformations have been observed for the TRAP1 tetramer in vitro: orthogonal, parallel, and antiparallel [22,73]. The conditions required for the adoption of or transition to a particular configuration are only predicted and remain unclear. A high-resolution MD-MD dimer–dimer interface has only been shown for the orthogonal structure (shown in the inset; adapted from Liu et. al., Biorxiv., 2020 [73]). Left and right sub-insets show interacting residues from protomer C (blue) to A (orange) and from A to C at the dimer–dimer interface, respectively.

Recently, TRAP1 was reported to form tetramers (dimer of dimers) [22], and it was proposed that the TRAP1 molecule exists in a dynamic equilibrium between a dimeric and a tetrameric state within mitochondria [22]. Analytical ultracentrifugation (AUC) with recombinant proteins further confirmed the existence of TRAP1 tetramers, which also seem to be stabilized in vitro by AMPPNP [73], a non-hydrolyzable structural homolog of ATP. Finally, cryo-EM analyses with purified proteins showed that the TRAP1 tetramer may adopt an orthogonal (butterfly), parallel, or antiparallel conformation (Figure 1) [73]. It should be noted that these observations are recent; any functional relevance of TRAP1 tetramers or for the potential transition between configurations remains unknown. Nevertheless, these observations are not entirely surprising when considering that crystallization of bacterial HtpG found the chaperone to exist as a dimer of dimers [74]. Similarly, HSP90 has also been reported to form such “oligomers” [75,76] under certain stimuli including elevated temperatures [77,78,79] and in the presence of non-ionic detergents or divalent cations [78,80].

Temperature-induced oligomerization of HSP90 is of particular interest in the context of TRAP1. This is because mitochondria operate close to 50 °C under physiological conditions, which is much higher than the 37 °C that is maintained in the adjacent cytosol [81]. To understand a physiological role of temperature-induced HSP90 oligomers, one study showed that self-oligomerized HSP90 under higher temperatures (>46 °C) readily binds to chemically unfolded dihydrofolate reductase (DHFR), a protein that could spontaneously refold by itself, to maintain it in a “folding-competent” state [79]. The binding of such a quaternary structure formed by HSP90 may actually provide an ideal environment for protein accommodation prior to folding and is consistent with a holdase function [82]. This hypothesis, while intriguing, definitely needs further experimental support. In the case of TRAP1, the existence of tetramers in “hot” mitochondria, the alterations in its asymmetry based on the availability of Mg^2+^ or Ca^2+^ ions, and a lack of significant proteome imbalance in TRAP1 KO cells [22] are consistent with the ability to adopt a holdase function in the mitochondrial environment. Additional experiments are needed to support or refute this hypothesis.

## 4. Cancer and Metabolic Rewiring

Cancers are generally characterized by a dramatic metabolic shift from OxPhos to aerobic glycolysis, a phenomenon that is commonly referred to as the Warburg phenotype [83,84,85,86]. The first indication that mitochondrial HSP90 is involved in cancer metabolism came from a study in 2012 reporting that this chaperone maintained metabolic homeostasis in neoplastic cells by inhibiting nutrient-sensing AMP kinase (AMPK), autophagy, and the unfolded protein response [87]. In the past 10 years, TRAP1 has been found to be highly expressed in a variety of neoplasms [24,47,48,49,50,51,52,53,54,55,56,57,59,60]. In 2013, an important observation provided a mechanistic basis for TRAP1 regulation of the balance between OxPhos and glycolysis in a variety of cell types [19]. More specifically, loss of TRAP1 led to an increase in mitochondrial respiration with a concomitant increase in oxygen-coupled ATP production, tricarboxylic acid (TCA) cycle activity, and fatty acid oxidation and the production of reactive oxygen species [19]. The re-introduction of TRAP1 restored this altered metabolic state to WT. Based on these observations and other supporting data, TRAP1 was proposed to act as a negative regulator of mitochondrial respiration, which exerted its effects via the inhibition of cytochrome C oxidase (Complex IV) and the mitochondrial pool of c-Src molecules [19]. Another independent study showed that TRAP1 directly binds to and inhibits the succinate dehydrogenase complex (SDH) [20], thereby downregulating mitochondrial respiration and the TCA cycle through a negative feedback generated by succinate accumulation [20,88,89,90]. Further, succinate accumulation inhibits hypoxia inducible factor (HIF) prolyl hydroxylation [91], stabilizing HIF1α [90] and creating a “pseudo-hypoxic” environment, which rewires cell metabolism towards glycolysis [90,92,93].

**Figure 2:**
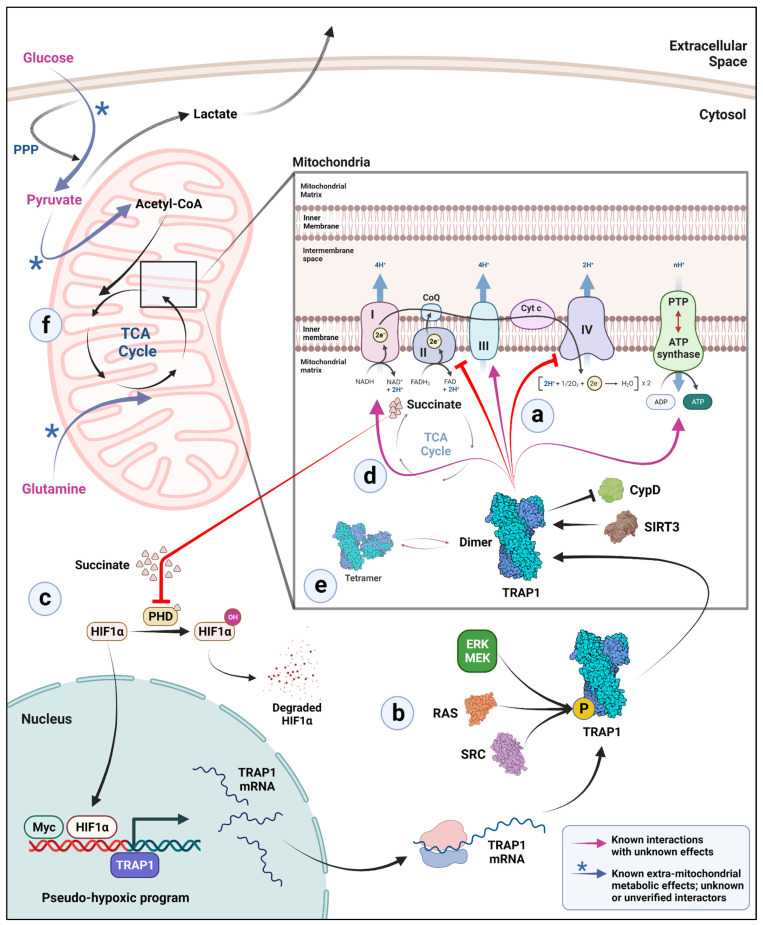
Potential mechanisms of TRAP1 participation in metabolic rewiring. (a) TRAP1 binds to and inhibits electron transport chain (ETC) complexes II and IV in the mitochondria. TRAP1 interacts with protein deacetylase SIRT3 in mitochondria and is reported to inhibit cyclophilin D (CypD), thereby preventing permeability transition pore (PTP) opening and inhibiting apoptosis due to cytochrome c release. (b) Further, TRAP1 activity is enhanced by phosphorylation via several pathways. Note that it remains unclear whether this happens before or after mitochondrial import of TRAP1. (c) ETC complex II inhibition by TRAP1 leads to succinate accumulation, which in turn inhibits prolyl hydroxylases in the cytosol to stabilize HIF1α. Stabilized HIF1α and Myc together activate a pseudo-hypoxic program, which further upregulates TRAP1 gene expression. (d) Inside mitochondria, TRAP1 also binds to ETC complexes I, III, and V (ATP synthase), but with unknown effects. (e) While TRAP1 tetramers exist alongside TRAP1 dimers in the mitochondrial matrix, determinants of the ratio of dimer to tetramer and any functional significance remain enigmatic. (f) TRAP1 presence and absence affect mitochondrial carbon preference. TRAP1 KO cells downregulate glucose- and pyruvate-derived carbon entry into the TCA cycle. A significant proportion of glucose is diverted to the pentose phosphate pathway (PPP) where it is used for the synthesis of NADPH reducing equivalents, perhaps to counter increased reactive oxygen species (ROS) that are characteristic of TRAP1 KO cells, and for the synthesis of ribose sugars. Pyruvate, upon decarboxylation, normally enters the TCA cycle and contributes to formation of acetyl-CoA, an important TCA cycle intermediate. In glycolysis, pyruvate is preferentially metabolized to lactate, generating NAD+ as a by-product of the reaction, at the expense of NADH. As with NADPH, increased levels of NADH provide more reducing equivalents to counter the increased ROS characteristic of TRAP1 KO. In contrast, TRAP1 KO cells utilize anaplerotic glutamine metabolism to maintain a functional TCA cycle by providing glutamine-derived carbon.

The early studies from Yoshida, Sciacovelli, and their colleagues [19,20] supported the hypothesis that TRAP1, by favoring a metabolic shift to glycolysis, is pro-tumorigenic. While this model is consistent with the reports highlighting the increased expression of TRAP1 in cancer, it is now clear that this molecule’s role in mitochondrial metabolism and function is likely more complex than originally predicted (Figure 2). Thus, a separate report proposed that TRAP1 was actually required for the maintenance of mitochondrial metabolism under nutrient-limiting conditions [21]. Further, Chae and coworkers suggested that TRAP1 does not inhibit SDH activity but instead promotes it to stabilize mitochondrial OxPhos [21]. Similarly, a very recent study reported that TRAP1 may also compete with the peptidyl-prolyl cis-trans isomerase cyclophilin D (CypD) for binding to the oligomycin sensitivity-conferring protein (OSCP) subunit of the ATP–synthase complex to increase its catalytic activity and to suppress the inhibitory effects of CypD [94]. Further, Park et al. recently reported that a dynamic interplay between TRAP1 and the histone deacetylase sirtuin 3 (SIRT3) not only promoted mitochondrial respiration but also maintained metabolic plasticity, stemness, and increased adaptation to stress in glioblastoma cells [23]. In this study, the loss of TRAP1 ameliorated the tumor-forming ability of glioblastomas in vivo [23]. A similar, but not identical, in vivo consequence of TRAP1 loss was reported in a TRAP1-deficient mouse model of breast cancer [61]. While TRAP1 was not required for tumor initiation, growth, or metastases induced by polyoma middle T-antigen, its loss was associated with a delay of tumor initiation in vivo and in inhibition of proliferation, migration, and invasion in vitro when compared to WT [61].

A mechanistic insight that can explain the physiological consequences of metabolic rewiring by TRAP1, both from a cancer and non-cancer perspective, remains elusive. This gap in our understanding may be partially attributed to the cell type or context-dependent effects of TRAP1 on metabolism and/or other aspects of mitochondrial dynamics. While its presence is certainly inhibitory for OxPhos in some scenarios [19,20,22], it is actually required for OxPhos maintenance in other contexts [21,23]. In an attempt to dissect common alterations in the central carbon metabolism of cells lacking TRAP1 (compared to isogenic WT cells), multiple cancer-derived cell lines were grown in otherwise non-limiting conditions but were limited as to the carbon sources that feed glycolysis and OxPhos [22]. Cells having different metabolic phenotypes, with or without TRAP1, were forced to rely on either glucose, pyruvate, or glutamine as the sole carbon source. Surprisingly, consistent among all the cell types considered, TRAP1-deficient cells were unable to support OxPhos with either glucose or pyruvate, instead relying on glutamine, which served as an anaplerotic molecule [95] to support the TCA cycle and OxPhos upon conversion to α-ketoglutarate in mitochondria. Confusingly, all these metabolic behaviors are pro-neoplastic [85,96,97,98]. This apparent paradox remains to be reconciled but may provide a basis for understanding the conflicting reports that TRAP1 may be pro- or anti-tumorigenic depending on cellular and environmental contexts. In another recent report consistent with the model proposed by Joshi et al. [22], glucose uptake and lactate production were also shown to be impaired in TRAP1-silenced colorectal cancer (CRC) cells exposed to hypoxic conditions [99].

## 5. Defining a TRAP1 Interactome

How TRAP1 ATP hydrolysis is coupled to its mitochondrial protein interactome has remained unclear. In a first attempt to address this question, Joshi and colleagues examined the TRAP1 interactome as a function of the chaperone’s ATPase activity [22]. Using a set of TRAP1 mutants displaying a 30-fold range of ATPase activity [69] and WT TRAP1 in a series of immunoprecipitation experiments followed by mass spectrometric analysis (IP-MS), the authors identified two distinct sets of interactors. The most abundant interactors were the mitochondrial chaperones mtHSP70/mortalin, HSP60, and prohibitin, whose binding to TRAP1 was not affected by TRAP1 ATPase activity. In contrast, a second, more diverse set of interactors, including the ATP synthase complex, translocases, proteins involved in mitochondrial membrane organization, and multiple subunits from mitochondrial electron transport chain complexes [22], displayed a strong negative correlation with the TRAP1 hydrolysis rate.

This second observation is in broad agreement with work done on HSP90 clients. In this case, HSP90 ATPase activity is inversely correlated with client binding and dwell time as part of the HSP90 complex [100]. Further, HSP90 mutants that bind ATP but cannot hydrolyze it demonstrate the strongest affinity for the HSP90 clients, HER2 and HSF1 [101,102]. These data suggest that TRAP1 interactors reflect the response of HSP90 clients to the chaperone’s ATPase activity. However, the lack of correlation between TRAP1 ATPase activity and interaction with other mitochondrial chaperones does not share obvious similarities with HSP90; the significance of this differential response requires further experimental study.

## 6. TRAP1 Inhibitors

Most inhibitors of the HSP90 family competitively bind to the N-terminal ATP pocket. This mode of action was exploited to create the first set of inhibitors for TRAP1, whose ATPase domain has homology with other members of the HSP90 family. However, since the mitochondrial membrane is impervious to traditional HSP90 inhibitors, a mitochondrial-targeting moiety such as one to four tandem repeats of cyclic guanidium or triphenylphosphonium (TPP) had to be added in order for these inhibitors to reach the mitochondrial matrix [33,103,104]. The first TRAP1 inhibitor was based on the benzoquinone ansamycin geldanamycin (specifically, 17-AAG), which was linked to a TPP moiety to create a “Gamitrinib” or a geldanamycin-based mitochondrial matrix inhibitor. Gamitrinibs accumulate in mitochondria and were shown to be anti-neoplastic in tumor xenografts and in mouse models of prostate cancer [105]. A similar TPP tagged derivative, SMTIN-P01, was also designed from PU-H71, a purine-based HSP90 inhibitor [106]. SMTIN-P01 also concentrated in mitochondria and was found to be cytotoxic to cancer cells [106]. Further, PU-H71-based TRAP1 inhibitors were shown to induce strong mitochondrial depolarization and apoptosis in acute myeloid leukemia cells [107]. PU-H71-based SMTIN-P01 was further modified with carbon spacers to create multiple analogs [108]. Of these, a 10-carbon spacer analog, SMTIN-C10, displayed both orthosteric and allosteric interactions with TRAP1 and changed its conformation from apo to closed state. While SMTIN-C10 increased TRAP1 ATPase activity, it perturbed TRAP1 function, decreased client protein levels, and exhibited anticancer activity both in vitro and in vivo [108]. These results are consistent with the previously discussed negative correlation between non-chaperone TRAP1 interactors and TRAP1 ATPase activity [22]. Importantly, to move forward, it will be necessary to determine systematically whether TRAP1 inhibitors phenocopy any of the consequences resulting from a stable TRAP1 KO.

While many TRAP1 inhibitors that have been reported or continue to be tested are linked to mitochondrial-targeting motifs such as TPPs, it is important to note that TPP itself is toxic to mitochondria [109]. TPP downregulates mitochondrial OxPhos; its non-specific effects likely would be additive to the consequences of specific TRAP1 inhibition. Moreover, such inhibitors *en route* to the mitochondrial matrix are expected to interact to some extent with the much more abundant HSP90 in the cytosol before even reaching the mitochondria [110]. As such, the possibility of substantial HSP90 inhibition with these TRAP1 inhibitors can never be ruled out. These issues were partially circumvented with the introduction of DN401, a BIIB021 [111] -derived pyrazolopyrimidine [110]. This molecule displayed increased TRAP1 selectivity over HSP90, exhibited potent in vivo anticancer activity, and lacked any mitochondrial-targeting motifs [110].

Such a continued rational design approach is likely to identify additional allosteric inhibitors of TRAP1, which either do not or poorly bind to HSP90. To this end, molecular dynamics simulations have been performed to understand the dynamic coordination between any two residues within the TRAP1 molecule as a function of the fluctuations between their distance [112,113]. Residues with high coordination were associated with low pair-distance fluctuations. Based on such simulations, a putative allosteric site responsible for structural reorganization of TRAP1 after ATP hydrolysis was identified in the middle domain of the chaperone. A pharmacophore model of this site was used to screen drug databases, and several TRAP1 selective inhibitors were identified [114]. These molecules specifically inhibit TRAP1 ATPase activity with minimal effects on HSP90 and were found to inhibit in vitro growth of malignant peripheral nerve sheath tumor (MPNST) cells [114]. A similar approach was used to identify a honokiol derivative, HDCA (honokiol bis-dichloroacetate), which was observed to bind to the same TRAP1 allosteric binding pocket, inhibiting its ATPase activity and its neoplastic potential in MPNST cells [115].

Recently, studies have explored whether TRAP1 inhibitors may be synergistic with other anticancer drugs. Gamitrinibs have been found to amplify the efficacy of inhibitors of mitogen-activated protein kinases in models of BRAFV600E melanoma and on drug-resistant melanoma cells [116]. Bromodomain and extraterminal (BET) family protein inhibitors JQ1 and OTX015 were also found to synergize with Gamitrinibs and to induce apoptosis in malignant glioma cells [117]. Gamitrinibs also augment the effect of histone deacetylase inhibitors in inducing apoptosis in patient-derived glioblastoma xenografts [118].

## 7. Conclusions

TRAP1 provides a link between mitochondrial homeostasis and metabolism. Although it is a member of the HSP90 family, which is well known for its roles in cellular proteostasis, cumulative studies over the last 20 years suggest that TRAP1 functions diverge from those of other HSP90 paralogs. It appears to be more closely related to the prokaryotic HtpG. TRAP1 does not bind to any known eukaryotic HSP90 co-chaperone and, unlike HSP90, it is essential neither in vitro nor in vivo. Further distinguishing TRAP1 from HSP90, a loss of TRAP1 does not significantly destabilize the mitochondrial proteome, but it impacts the mitochondrial matrix structure and modulates mitochondrial metabolism to maintain metabolic plasticity. Unlike other members of the HSP90 family, TRAP1 molecules readily form tetramers in the “hot” mitochondrial matrix. Whether this is a direct consequence of elevated temperature and whether these tetramers may promote assembly and/or stabilization of large macromolecular structures common to mitochondria, their functional relevance, dynamics, and regulation remain unknown.

Even with all these questions and challenges, understanding the TRAP1 function and how it integrates dynamic alterations in the mitochondrial structure and cell metabolism, survival, and growth from neoplastic and non-neoplastic perspectives is a rapidly evolving field that retains great interest, especially in light of the emerging importance of mitochondria in many unexpected cellular processes. Continued deep analysis of TRAP1 dynamics, interactors, and functions is likely to prove rewarding in this context.
